# Critical role of NLRP3-caspase-1 pathway in age-dependent isoflurane-induced microglial inflammatory response and cognitive impairment

**DOI:** 10.1186/s12974-018-1137-1

**Published:** 2018-04-17

**Authors:** Zhi Wang, Shiyu Meng, Lin Cao, Ying Chen, Zhiyi Zuo, Shuling Peng

**Affiliations:** 1 Department of Anesthesiology, Sun Yat-Sen Memorial Hospital, Sun Yat-Sen University, Guangzhou, 510289 Guangdong China; 2 Laboratory of RNA and Major Diseases of Brain and Heart, Sun Yat-Sen Memorial Hospital, Sun Yat-Sen University, Guangzhou, 510120 China; 3 Department of Anesthesiology, University of Virginia, Charlottesville, USA

**Keywords:** Aging, Isoflurane, Neuroinflammation, NOD-like receptor protein 3 inflammasome, Postoperative cognitive dysfunction

## Abstract

**Background:**

Elderly patients are more likely to suffer from postoperative cognitive dysfunction (POCD) after surgery and anesthesia. Except for declined organ function, the particular pathogenesis of POCD in elderly patients remains unknown. This study is carried out to determine the critical role of the NOD-like receptor protein 3 (NLRP3)-caspase-1 pathway in isoflurane-induced cognitive impairment.

**Methods:**

Young (6–8 months old) and aged (14 months old) healthy male C57BL/6 mice were exposed to 1.5% isoflurane for 2 h. Some mice received intraperitoneal injection of Ac-YVAD-cmk (8 mg/kg), a specific inhibitor of caspase-1, 30 min before the isoflurane exposure. Morris water maze test was carried out 1 week after the isoflurane anesthesia. Brain tissues were harvested 24 h after the isoflurane anesthesia. Western blotting was carried out to detect the expression of NLRP3, interleukin (IL)-1β, and IL-18 in the hippocampus. Mouse microglial cell line BV-2 and primary microglial cultures were primed by lipopolysaccharide for 30 min before being exposed to isoflurane. NLRP3 was downregulated by RNA interference.

**Results:**

Compared to young mice, aged mice had an increased expression of NLRP3 in the hippocampus. Isoflurane induced cognitive impairment and hippocampal inflammation in aged mice but not in young mice. These effects were attenuated by Ac-YVAD-cmk pretreatment (*P* < 0.05). Isoflurane activated NLRP3-caspase-1 pathway and increased the secretion of IL-18 and IL-1β in cells pretreated with lipopolysaccharide but not in cells without pretreatment. Downregulation of NLRP3 attenuated the activation of NLRP3 inflammasome by isoflurane.

**Conclusions:**

NLRP3 priming status in aged mouse brain may be involved in isoflurane-induced hippocampal inflammation and cognitive impairment.

**Electronic supplementary material:**

The online version of this article (10.1186/s12974-018-1137-1) contains supplementary material, which is available to authorized users.

## Background

Human aging has been viewed as functional decline and reduced reserve in multiple systems [1, 2]. Elderly patients are more likely to suffer from postoperative cognitive dysfunction (POCD), which includes memory, concentration, or attention impairment, and is associated with increased mortality [3, 4]. Although POCD is a very significant clinical syndrome, its etiology and mechanism remain largely unknown.

Neuroinflammation has been suggested to play a critical role in the development of POCD [4]. Clinical and experimental evidence has shown that anti-inflammation treatment could reduce POCD [5–7]. Age-related inflammation is characterized by a chronic mild inflammation called “inflammaging” [1, 8], which negatively impacts central nervous system (CNS) function [9]. In aged brain, microglia develop a “priming” phenotype and an overactive inflammatory status, which consequently impairs cognitive function [7, 10–12].

Volatile anesthetics, such as isoflurane, have been shown to induce inflammatory response and increase caspase-3 activation in the aged rat brain [13, 14]. Our previous study has shown that lidocaine attenuates cognitive impairment after isoflurane anesthesia in aged rat by anti-neuroinflammation [15]. In addition, isoflurane does not influence the cognitive function of interleukin (IL)-1β-deficient mice [15]. Notably, young animals develop little neuroinflammation or cognitive impairment after isoflurane exposure [16]. These results suggest the critical role of neuroinflammation in isoflurane-induced cognitive impairment in aged animals.

Known as one of the innate immune sensors, the NOD-like receptor protein 3 (NLRP3) inflammasome composes of NLRP3, apoptosis-associated speck-like protein containing a caspase recruitment domain (ASC), and caspase-1. This inflammasome regulates caspase-1 activation that controls the maturation and secretion of IL-1β and IL-18 [17–19]. Compared to young mice, canonical NLRP3 inflammasome activation is linked to inflammation-induced cognitive function decline and neuropathological changes with aging [20]. Ablation of each component of NLRP3 inflammasome protects cognitive function from age-related neuroinflammation [20] and neurodegeneration, such as in Alzheimer’s disease [21, 22]. NLRP3 inflammasome activation has also been suggested to be the mechanism for the neurotoxicity and cognitive impairment induced by polybrominated diphenyl ethers, materials used widely in industry [23]. Interestingly, canonical NLRP3 inflammasome activation takes two steps [24]. First, priming by up-regulation of NLRP3 is likely via nuclear factor-κB (NF- κB) pathway to increase *NLRP3* transcription. The deubiquitination of NLRP3 protein allows the protein to be assembled with the components of NLRP3 inflammasome, which completes the priming process [25]. The second step involves activating the NLRP3 inflammasome by diverse “danger signals” to induce caspase-1 activation. Notably, NLRP3 inflammasome can be activated by damage-associated molecular patterns (DAMPs) and reactive oxygen species [25, 26]. However, the processes of the priming and activation are largely unknown (Additional file 1).

Based on the above discussion, we hypothesize that aging-associated NLRP3 priming makes the aged brain vulnerable for cognitive dysfunction after isoflurane exposure. To test our hypothesis, we performed animal and cell culture studies.

## Methods

### Ethics statement

All animal experiments were performed in accordance with current Chinese regulations and standards regarding the use of laboratory animals, and approved by the animal ethics committee of Sun Yat-Sen University.

### Animal groups

Six to 8-month-old male C57BL/6 mice were randomly assigned to two groups: (1) group YC (young mice not being exposed to isoflurane or any drugs), or (2) group Y-ISO (young mice exposed to isoflurane). Fourteen-month old male C57BL/6 mice were randomly assigned to four groups: (1) group AC (aged mice not being exposed to isoflurane or any drugs), (2) group A-ISO (aged mice exposed to isoflurane), (3) group A-ISO-cmk (Ac-YVAD-cmk administered before aged mice were exposed to isoflurane), or (4) group A-ISO-PBS (solvent administered before aged mice were exposed to isoflurane). Each group had 21 mice: 15 mice were subjected to Morris water maze test and 6 mice were sacrificed for brain tissue preparation.

### Animal treatment

The mice received isoflurane anesthesia in a gas-tight chamber prefilled with 1.5% isoflurane in 100% O_2_. The chamber was continuously gassed with 1.5% isoflurane in 100% O_2_ at 1.5 L/min. Mice were in this chamber for 2 h [16]. Isoflurane concentration was continuously monitored by sampling the exhaust gases with a Datex-Ohmeda ULT-SV analyzer (Madison, USA). After anesthesia, the mice were supplied with 100% O_2_ for emergency.

Ac-YVAD-cmk (Sigma-Aldrich, Darmstadt, Germany), an inhibitor of NLRP3-caspase-1, was first dissolved in dimethylsulphoxide (DMSO) and then diluted with phosphate-buffered saline (PBS). Group A-ISO-cmk or group A-ISO-PBS received intraperitoneal administration of 8 mg/kg Ac-YVAD-cmk or its solvent (PBS + DMSO), respectively, 30 min before isoflurane anesthesia [27]. The mice in the group YC and AC received 100% O_2_ for 2 h in an identical chamber. Mouse temperature was maintained at 37 ± 0.5 °C by an animal heating pad during the anesthesia procedure.

### Morris water maze (MWM)

1 week after isoflurane exposure, animals were subjected to MWM to test their spatial learning and memory as described [28]. The MWM assesses spatial learning and memory ability of animals. The MWM consists of a hidden platform located in one of the quadrants of a 1-m diameter circular pool. Mice were placed in a quadrant other than the quadrant where the hidden platform was located and then the time taken (latency) for the mouse to find the hidden platform was recorded. Each mouse was released from three different quadrants and was allocated 120 s to find the platform. If they were unable to do so, the mouse was then guided to the platform and allowed to remain on the platform for 30 s. The mice were trained in five consecutive days. The platform was removed on the sixth day, and mice were allowed to swim for 120 s to test their memory (platform-crossing times and target quadrant traveling time). The escape latency to reach the platform during the 5 days of training as well as the target quadrant traveling time, platform-crossing times, and the average swimming speed on the sixth day were used to reflect the performance of mice in MWM. Relative average escape latency was the average escape latency during the 5 days of training. The final result of this parameter was calculated in the following way. First, the average escape latency in the 5 days of training was calculated for each mouse. These data were then normalized by the corresponding average escape latency of the group YC (for all groups of young mice) or group AC (for all groups of aged mice).

### Cell culture and treatment

The BV-2 mouse microglial cell line was purchased from Bio-platform (Guangzhou, China). The cells were grown in DMEM/F-12 containing 10% fetal bovine serum (FBS), 10 μg/mL streptomycin, and 10 U/mL penicillin at 37 °C under 5% CO_2_ and 95% air. BV-2 cells from passages 2 to 4 were used in experiments.

To prepare primary microglial cultures, 1- to 3-day old C57BL/6 mice were sacrificed. The whole brain was harvested on ice and digested in 0.125% trypsin and 12.5 kU/mL DNase I at 37 °C for 20 min. Pooled cells prepared from two mice were planted in one 75 cm^2^ cell culture flasks for 14 days. The floating microglial cells over the mixed glial cultures were collected and seeded in 6-well plates. The purity of the primary microglial cultures was > 95% as determined by flow cytometry staining for CD11b.

BV-2 cells and primary microglial cultures were incubated with lipopolysaccharide (LPS) or control culture medium. The cells were subsequently exposed to or were not exposed to isoflurane in 5% CO_2_ and 95% air for 6 h in a gas-tight chamber at 37 °C. During isoflurane exposure, the isoflurane concentration was continuously monitored by a Datex-Ohmeda ULT-SV analyzer. Four treatment groups were studied: control, ISO, LPS (NLRP3-primed), LPS (NLRP3-primed) + ISO.

### Western blotting

After transcardial perfusion with ice-cold PBS under deep anesthesia, the hippocampus was harvested at 24 h after the isoflurane exposure for the determination of NLRP3, IL-1β, and IL-18 expression. The BV-2 cells were harvested immediately after the isoflurane treatment. Briefly, hippocampus were removed immediately on ice and frozen in liquid nitrogen. Frozen tissues were lysed in Strong RIPA lysis buffer (Beyotime, Shanghai, China) with 1% Protease Inhibitor Cocktail (Biotool, Houston, USA) for 30 min on ice. The solution was centrifuged for 20 min at 12000 rpm at 4 °C, and the supernatant was used for western blotting. BV-2 cells and primary microglial cultures were harvested immediately after isoflurane exposure. Cells were lysed in RIPA lysis buffer (Beyotime, Shanghai, China) with 1% Protease Inhibitor Cocktail for 30 min on ice. The solution was centrifuged for 20 min at 12000 rpm at 4 °C and the supernatant was used for western blotting.

Proteins from lysed tissue and cells were separated by SDS-PAGE and transferred to polyvinylidene difluoride membranes (Millipore, USA). The membrane was blocked in 5% skim milk dissolved in Tris-buffered saline containing 0.1% Tween-20 (TBST) for 1 h and incubated overnight at 4 °C with specific antibody against NLRP3 (1:1000, Cell Signaling Technology, USA), IL-1β (1:1000, Cell Signaling Technology, USA), IL-18 (1:1000, Abcam, UK), caspase-1 p45 (1:500, Santa Cruz, CA), caspase-1 p20 (1:500, Santa Cruz, CA), ASC (1:500, Santa Cruz, CA), cleaved caspase-3 (1:1000, Abcam, UK), Iba-1 (1:1000, Abcam, UK), or glyceraldehyde-3-phosphate dehydrogenase (GAPDH) (1:10000, Abcam, UK), respectively. The membranes were then incubated with an HRP-conjugated secondary antibody. The volumes of protein bands were measured, and the values of protein bands were normalized by these of GAPDH from the same samples.

### RNA isolation and real-time PCR assay

Total RNA was extracted with TRIzol reagent (TaKaRa, Japan). Reverse transcription was performed with PrimeScript RT reagent Kit (TaKaRa, Japan) according to the manufacturer’s instructions. For real-time PCR analysis, the resultant cDNA products were amplified using SYBR Green qPCR Master Mix (Biotool, USA) in triplicates. Primer sequences of the genes analyzed were NLRP3 5′-ATTACCCGCCCGAGAAAGG-3′ and 5′-TCGCAGCAAAGATCCACACAG-3′; GAPDH 5′ –GGTGAAGGTCGGTGTGAACG-3′ and 5′-CTCGCTCCTGGAAGATGGTG-3′.

### Enzyme-linked immunosorbent assay (ELISA)

The IL-1β secreted into cell supernatants was quantitated by ELISA (Biolegend, San Diego, CA, USA) according to the manufacturer’s instruction. The absorbance was measured at 450 and 570 nm using a micro-plate reader.

### Cell viability

The cell cytotoxicity assay was performed using Cell Counting Kit-8 (CCK-8) (Dojindo Molecular Technologies, Shanghai, China) according to the manufacturer’s instructions. Approximate 5.0 × 10^4^ cells were plated in 96-well plates overnight. Cells were subjected to 1 μg/mL LPS for 0.5 h before the exposure to 4% isoflurane for 6 h. To assay the cytotoxicity, 10 μL of CCK-8 solution was added to each well of the plate 0 and 12 h after isoflurane exposure. The absorbance was recorded at 450 nm on a micro-plate reader. The data were collected from three independent experiments.

### RNA interference assay

RNA oligo nucleotides prepared by GenePharma (Shanghai, China) were transfected into BV-2 cells using lipofectamine RNAiMax (Invitrogen, Carlsbad, USA) according to the manufacturer’s instructions. The NLRP3 siRNA sequences used were 5′-GGCGAGACCUCUGGGAAAATT-3′ and 5′-UUUUCCCAGAGGUCUCGCCTT-3′.

### Immunoprecipitation

BV-2 cells were used in immunoprecipitation. Total protein lysate from BV-2 cells was prepared in immunoprecipitation buffer. Five hundred microgram protein was mixed with 2.5 μg anti-NLRP3 antibody, or IgG. The mixture was incubated on a rotating shaker at 4 °C for 8 h. Beads (Santa Cruz, CA, USA) were added to the mixture and incubated at 4 °C overnight. The beads were collected by centrifugation at 2500 rpm for 5 min at 4 °C, and then washed 3–5 times by immunoprecipitation buffer. Sample loading buffer at 5× was added to the beads before being boiled for 10 min. The supernatant was collected and used in western blotting.

### Statistical analysis

Values were presented as the mean ± SD or in individual value. Statistical analysis was performed with SPSS 22.0 (SPSS Inc., Chicago, IL, USA). Statistical comparisons among groups were analyzed by one-way analysis of variance (ANOVA), and post hoc comparisons were conducted by the Bonferroni test or by Dunnett method if the homogeneity of variance was not met. Escape latency during the training of water maze was analyzed by two-way repeated measures ANOVA for the comparisons between different groups. Platform crossing times were analyzed by ANOVA on ranks. Results were considered statistically significant at *P* < 0.05.

## Results

### Isoflurane induced age-related cognitive impairment

**Fig. 1:**
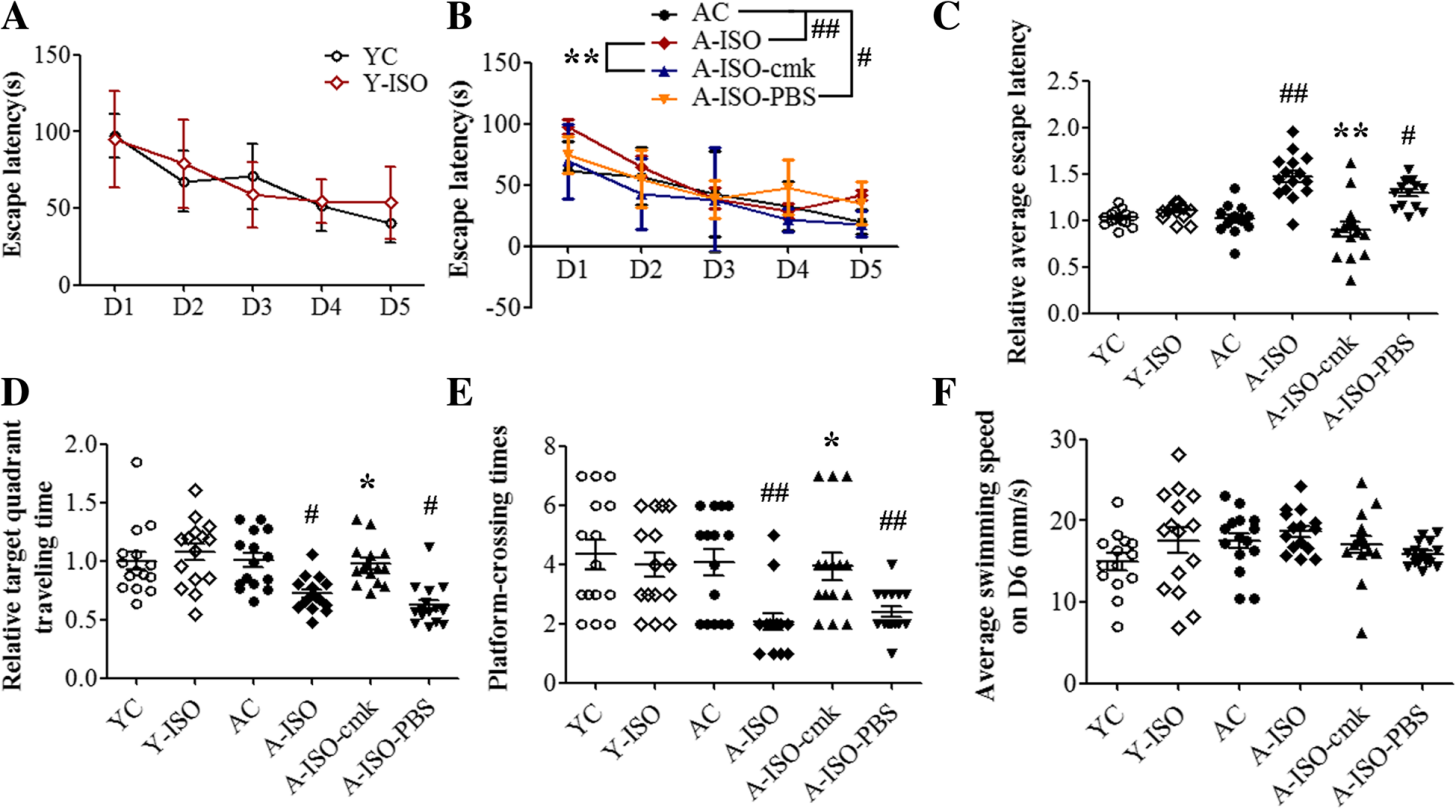
Isoflurane induced age-related cognitive impairment. Young (6–8 months old) and aged (14 monts old) healthy male C57BL/6 mice were exposed to 1.5% isoflurane for 2 h. Some aged mice received intraperitoneal injection of 8 mg/kg Ac-YVAD-cmk 30 min before the isoflurane exposure. YC = blank control of young mice; Y-ISO = young mice exposed to isoflurane; AC = blank control of aged mice; A-ISO = aged mice exposed to isoflurane; A-ISO-cmk = Ac-YVAD-cmk administered before aged mice exposed to isoflurane; A-ISO-PBS = solvent of Ac-YVAD-cmk (PBS+ less than 1% DMSO) administered before aged mice exposed to isoflurane. Morris water maze test was carried out 1 week after the isoflurane anesthesia. **a**, **b**, and **c** Escape latency to reach the platform. **d** Target quadrant traveling time. Normalization of the target quadrant traveling time of group YC and Y-ISO was by the mean of group YC, and group AC, A-ISO, A-ISO-cmk, and A-ISO-PBS by the mean of group AC. **e** Platform-crossing times. **f** Average swimming speed on the sixth day. All results are mean ± SD (*n* = 15). #*P* < 0.05 and ##*P* < 0.01 compared with the corresponding data of group AC. *P < 0.05 compared with the corresponding data of group A-ISO

No animal died or was apneic during the anesthesia or the intended observation period after the anesthesia. In MWM, the escape latency of each training day and the relative average escape latency of five training days were used to indicate the learning ability. Significant differences in escape latency during the whole training phase were observed between group AC and group A-ISO, group A-ISO-cmk and group A-ISO, group A-ISO-cmk and group A-ISO-PBS (Fig. 1a, b). The relative average escape latency of group A-ISO in five training days was significantly longer than group AC. This increase was attenuated by the pretreatment with the caspase-1 inhibitor Ac-YVAD-cmk (Fig. 1c). In the probe trail, target quadrant traveling time and the number of platform-crossing indicate the memory activities. Compared to group AC, the time in the target quadrant and frequency to cross the platform were significantly decreased in group A-ISO and group A-ISO-PBS. This decrease was reversed by Ac-YVAD-cmk pretreatment (Fig. 1d, e). No significant difference was observed in the average swimming speed on the sixth day among the groups (Fig. 1f). Different from the situation in aged mice, isoflurane did not have an effect on the performance of young mice in MWM (Fig. 1a–f). These results indicate that 1.5% isoflurane anesthesia for 2 h leads to cognitive impairment in the aged mice and that this impairment can be attenuated by Ac-YVAD-cmk pretreatment.

### Isoflurane induced NLRP3 inflammasome activation in aged brain

**Fig. 2:**
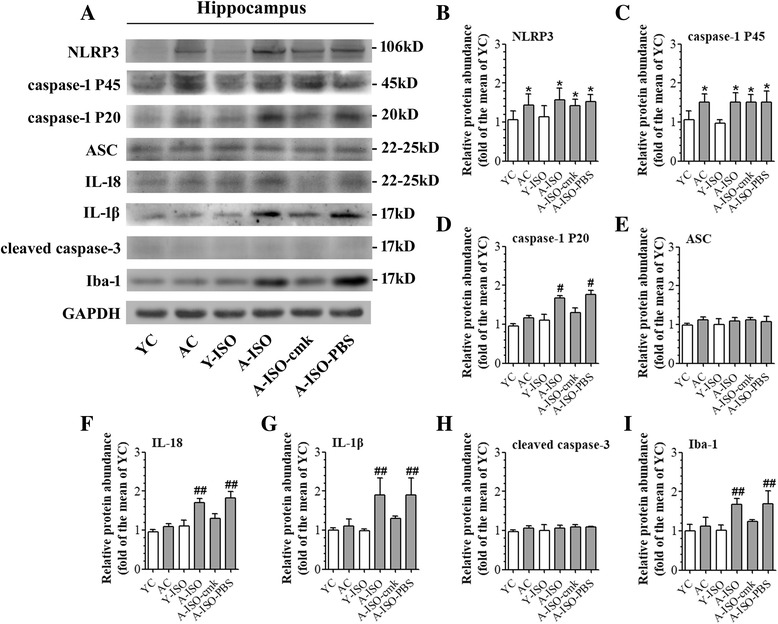
Contribution of NLRP3 inflammasome activation to isoflurane-induced age-related neuroinflammation. Hippocampus was harvested at 24 h after the anesthesia. **a** Representative western blot images of NLRP3, caspase-1 P45, caspase-1 P20, ASC, IL-18, IL-1β, cleaved caspase-3, and Iba-1. **b**–**i** Graphic presentation of abundance of each protein. All values are expressed as fold changes over the mean values of group YC and are presented as mean ± SD (*n* ≥ 3). *P < 0.05 compared with the corresponding data of group YC. #*P* < 0.05 and ##*P* < 0.05 compared with the corresponding data of group AC

Compared to group YC, group AC had an increased expression of NLRP3 in the hippocampus. The levels of caspase-1 P20, cleaved IL-18, cleaved IL-1β, and Iba-1 were upregulated after isoflurane treatment in the aged mice, which was reversed by Ac-YVAD-cmk pretreatment (Fig. 2a–g, i). These results indicate that the NLRP3 inflammasome activation may contribute to isoflurane-induced age-related hippocampal inflammation. The levels of cleaved caspase-3 were not upregulated in the hippocampus of aged mice no matter whether they were exposed to isoflurane (Fig. 2a, h).

### NLRP3 inflammasome priming was induced by LPS stimulation

**Fig. 3:**
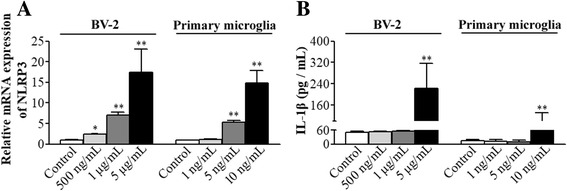
NLRP3 priming was induced by LPS stimulation. Different doses of LPS were added to the cell culture media of BV-2 cells or primary microglial cultures for 30 min. **a** The mRNA of NLRP3 was quantified by real-time qPCR immediately after LPS stimulation. Values are expressed as fold changes over the mean values of blank control. **b** IL-1β concentration in the supernatant was measured 6 h later. All results are presented as mean ± SD (*n* ≥ 3). *P < 0.05 and **P < 0.01 compared with the corresponding data of blank control

To determine the characteristics of LPS-induced priming of NLRP3 inflammasome pathway, BV-2 cells and primary microglial cultures were incubated with various doses of LPS. LPS induced a dose-dependent increase of NLRP3 mRNA. Interestingly, this increase was induced by much lower doses of LPS in the primary microglial cultures than in the BV-2 cells. Nevertheless, most doses that induced NLRP3 mRNA expression did not increase the concentration of IL-1β in the culture medium of the BV-2 cells and primary microglial cultures. The increase of IL-1β happened only when a very high concentration (25 μg/mL in BV-2 cells or 10 ng/mL in primary microglial cultures) of LPS was used (Fig. 3). These results suggest that those concentrations of LPS that increased NLRP3 mRNA but did not increase IL-1β induced a priming status of the microglial cells.

### NLRP3 priming was necessary in isoflurane-induced NLRP3 inflammasome activation

**Fig. 4:**
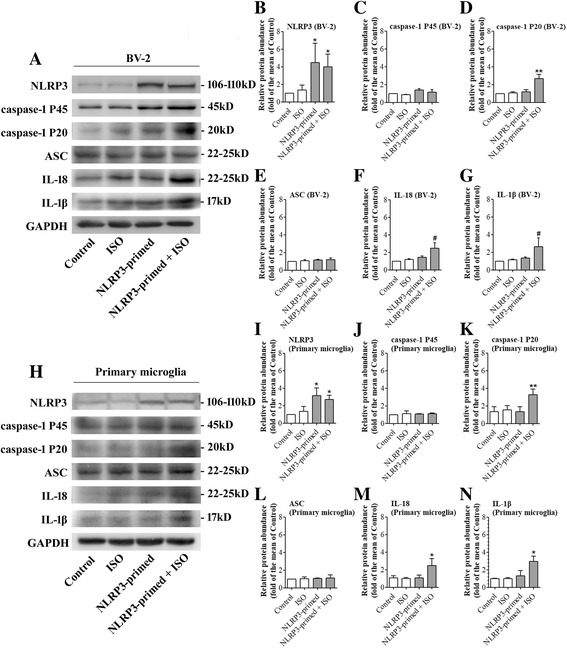
Isoflurane-induced NLRP3 inflammasome activation after priming. BV-2 microglia were exposed to 4% isoflurane for 6 h in the cells primed with or without 1 μg/mL LPS. Primary microglial cultures were exposed to 2% isoflurane for 6 h in the cells primed with or without 5 ng/mL LPS. Control = blank control; ISO = Isoflurane exposure; NLRP3-primed = LPS stimulation; NLRP3-primed+ ISO = NLRP3-primed + isoflurane exposure. **a** Western blot images of NLRP3, caspase-1 P45, caspase-1 P20, ASC, IL-18, and IL-1β from BV-2 cells. **b**–**g** Graphic presentation of abundance of each protein in BV-2 cells. **h** Western blot images of NLRP3, ASC, caspase-1 P45, and caspase-1 P20, IL-18, and IL-1β. **i**–**n** Graphic presentation of abundance of each protein in primary microglia. Values are expressed as fold changes over the mean values of control and are presented as mean ± SD (*n* ≥ 3). *P < 0.05 and **P < 0.01 compared with the corresponding data of group control. #*P* < 0.05 and ##*P* < 0.01 compared with the corresponding data of group NLRP3-primed

**Fig. 5:**
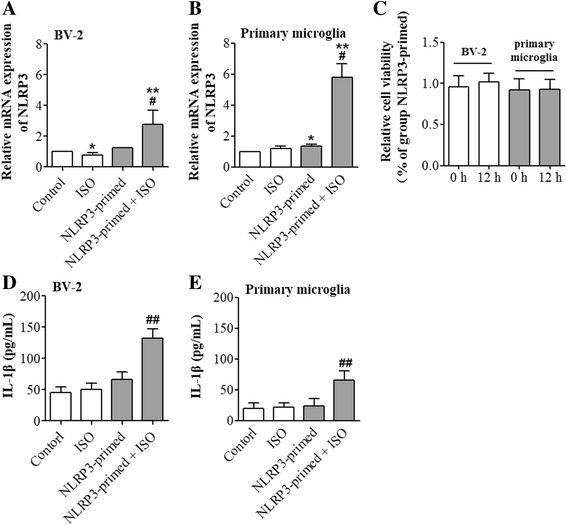
NLRP3 priming was necessary in isoflurane-induced IL-1β production. BV-2 cells primed with or without 1 μg/mL LPS were exposed to 4% isoflurane for 6 h. Primary microglial cultures primed with or without 5 ng/mL LPS were exposed to 2% isoflurane for 6 h. Control = blank control; ISO = isoflurane exposure; NLRP3-primed = LPS stimulation; NLRP3-primed + ISO = NLRP3-primed + isoflurane exposure. **a** The mRNA of NLRP3 in BV-2 cells. Values are expressed as fold changes over the mean values of blank control and are presented as mean ± SD (*n* = 6). **b** IL-1β concentration in the supernatant of BV-2 cells. **c** Viability of NLRP3-primed cells at 0 and 12 h after isoflurane exposure. Values are expressed as fold changes over the mean values of NLRP3-primed cells and are presented as mean ± SD (*n* = 3). **d** The mRNA of NLRP3 in primary microglial cultures. Values are expressed as fold changes over the mean values of blank control and are presented as mean ± SD (*n* = 3). **e** IL-1β concentration in the supernatant of primary microglial cultures. Values are expressed as fold changes over the mean values of control and are presented as mean ± SD (*n* = 3). *P < 0.05 and **P < 0.01 compared with the corresponding data of group control. #*P* < 0.05 and ##*P* < 0.01 compared with the corresponding data of group NLRP3-primed cells

Consistent with the data presented in Fig. 3, 1 μg/mL LPS increased the expression of NLRP3 in BV-2 cells. Similarly, the combination of LPS and isoflurane also increased the expression of NLRP3. After NLRP3 priming by 1 μg/mL LPS, 4% isoflurane treatment induced a significant IL-1β and IL-18 production (Figs. 4a, b and 5a). Interestingly, 4% isoflurane alone could not increase IL-1β or IL-18 production (Fig. 4a, h). Similarly, isoflurane-induced caspase-1 activation only in NLRP3-primed in BV-2 cells, indicating that NLRP3 priming is necessary in isoflurane-induced NLRP3 inflammasome activation (Figs. 4a–g and 5d). To determine whether the findings from the immortalized BV-2 cell line were reproducible in the primary microglial cultures, we performed a similar experiment. In primary microglial cultures, the combination of LPS and isoflurane also increased the expression of NLRP3 (Figs. 4h, i and 5b). After NLRP3 priming by 5 ng/mL LPS, 2% isoflurane treatment significantly increased IL-18 and IL-1β production, while no significant caspase-1 activation or IL-1β production was induced by isoflurane alone (Figs. 4h–n and 5e). We further investigated the effect of isoflurane on the viability of BV-2 cells and primary microglial cultures by using CCK-8 cell viability assay. No significant differences in the cell viability were observed between cells that were harvested immediate after LPS priming and primed cells that were harvested 12 h after an exposure to isoflurane for 6 h (Fig. 5c).

### NLRP3 knock-down reduced isoflurane-induced NLRP3 inflammasome activation

**Fig. 6:**
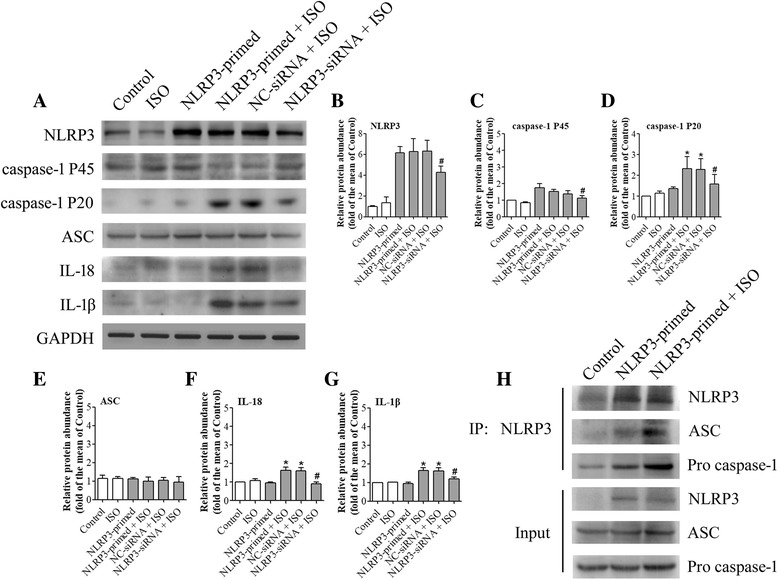
NLRP3 knock-down reduced isoflurane-induced NLRP3 inflammasome activation. Cells were transfected with NLRP3 siRNA or negative control siRNA 24 h before NLRP3 priming and exposure to 4% isoflurane for 6 h. NC-siRNA + ISO = negative control of siRNA before NLRP3 priming and isoflurane exposure; NLRP3-siRNA + ISO = NLRP3 siRNA before NLRP3 priming and isoflurane exposure. **a** Western blot images of NLRP3, caspase-1 P45, caspase-1 P20, ASC, IL-18, and IL-1β. **b**–**g** The graphic presentation of each protein abundance of Fig. 6a. **h** Whole cell protein samples from BV-2 cells were harvested immediately after isoflurane treatment. Immunoprecipitation was performed by using an anti-NLRP3 antibody and was immunoblotted for NLRP3, ASC, and pro-caspase-1. Values are expressed as fold changes over the mean values of NLRP3-primed cells and are presented as mean ± SD (*n* = 6). *P < 0.05 compared with the corresponding data of group control. #*P* < 0.05 compared with the corresponding data of NLRP3-primed + ISO

To determine the role of NLRP3 in the isoflurane effects, NLRP3 protein was knocked-down in the BV-2 cells. This downregulation significantly reduced the increase of caspase-1 P20 and P45 caused by the priming and isoflurane exposure (Fig. 6a–g). These results suggest that downregulating NLRP3 protein inhibits caspase-1 activation, an event that indicates NLRP3 inflammasome activation.

### NLRP3 inflammasome assembly might be enhanced by isoflurane

Isoflurane appeared to increase the amount of pro-caspase-1 and ASC associated with NLRP3 (Fig. 6h). ASC is an adaptor protein for NLRP3 inflammasome [25]. Isoflurane did not change the input of ASC and pro-caspase-1 (Fig. 6h). These results suggest that isoflurane increases NLRP3 inflammasome assembly.

## Discussion

Elderly patients have a higher incidence rate of POCD after surgery and anesthesia [29]. The precise mechanism for the phenomenon remains unknown. Compared to young mice, the priming status of NLRP3 inflammasome has been found in the microglia of aged brain. Canonical NLRP3 inflammasome activation is reported to be linked to inflammation-induced cognitive function decline in aging [20] and Alzheimer’s disease [21]. Our study was performed to determine the effect of isoflurane on NLRP3 inflammasome activation.

In our study, we showed that aged mice had poorer learning and memory function after isoflurane anesthesia. This effect was not apparent in young mice. In addition, we found that NLRP3 inflammasome was in primed status in the hippocampus of aged mice but such a status did not exist in young mice. An NLRP3-caspase-1 pathway inhibitor could attenuate the cognitive impairment caused by isoflurane anesthesia in the aged mice. These results suggest that NLRP3 may play a role in the impairment of learning and memory function of aged mice caused by isoflurane anesthesia. To support this finding, we found that isoflurane could induce NLRP3 inflammasome activation in both BV-2 cells and primary microglial cultures after they were primed. We further observed that isoflurane might induce NLRP3 inflammasome activation by increasing NLRP3 inflammasome assembly. These findings suggest a potential target for reducing isoflurane-induced, age-related cognitive dysfunction.

Isoflurane, a widely used inhalational anesthetic, has been reported to impair the cognitive function in rodents, especially in the aged rodents [13, 15, 16]. Since neuroinflammation has been found to play an important role in cognitive dysfunction after surgery or anesthesia [4, 13, 30], anesthetic effects on neuroinflammation have been a focus of research. Isoflurane alone has been reported to have no or a small effect on cytokine expression in microglia under control condition [31]. In addition, treatment with 2% isoflurane alone on primary neuron cultures for 6 h did not enhance the transcription binding activity of NF-kB [32] but increased the death and NF-kB transcriptional activities in SH-SY5Y neuroblastoma cells after oxygen-glucose deprivation [33]. One minimum alveolar concentration (the concentration at which 50% of animals have no motor response to painful stimuli) of isoflurane in 14-month-old mice is 1.53 +/− 0.14% [34]. According to our previous study and reports from others, 1.5% isoflurane treatment for 2 h impaired the cognitive functions of rodents, including learning and memory function and anxiety-related behaviors, while the blood gas analysis demonstrated no hypoxia or acidosis immediately after the isoflurane exposure [16, 35, 36]. Our previous study showed that IL-1β played a critical role in isoflurane-induced cognitive dysfunction [13]. In addition to IL-1β, isoflurane also increased IL-6 and tumor necrosis factor (TNF)-α in the brain [37]. Consistent with these previous studies, a higher level of proinflammatory cytokines was observed in the aged hippocampus and the aged mice had learning and memory dysfunction after isoflurane anesthesia. Isoflurane did not induce learning and memory impairment in young mice. These findings suggest that aged brain have an overactive inflammatory response after isoflurane exposure, which may be a mechanism for cognitive impairment.

Aging is known to impair microglial functions with increased susceptibility to pro-inflammatory activation, thereby promoting aging-related neurodegeneration [38]. Previous studies have demonstrated that a common start of neuroinflammation is microglial activation and the subsequent inflammatory cascade, which lead to micro-environment for central nervous system disorder and ultimately affect cognitive function [39, 40]. Microglia secrete pro-inflammatory cytokines, like IL-1β and TNF-α, after being stimulated by ischemia or infection [39, 41]. These pro-inflammatory cytokines, together with astrocytes, neurons, and oligodendrocytes, regulate neuroinflammation. In the aging brain, it has been proposed that microglia may be in a primed status to be activated [11]. A consequence of microglial priming with age is a hyperactive response to an immune challenge with an exaggerated pro-inflammatory response.

The role of the NLRP3 inflammasome in neurodegenerative diseases has recently been investigated. The NLRP3 inflammasome activation leads to secreting inflammatory factors, like IL-1β and IL-18, in the brain. Researches have indicated that functional NLRP3 inflammasome activation is limited to the microglial compartment in the mouse brain [42]. Furthermore, The APP/PS1 mice (a mouse model for Alzheimer’s disease) with NLRP3 knockout are protected from spatial memory impairment and have a decreased Aβ plaque burden [43]. Known as a major sensor of age-related accumulation of DAMPs and an upstream regulator that controls caspase-1-mediated pro-inflammatory state in aging [38, 44], it is important to identify how NLRP3 inflammasome expression and activation occurred in aged brain after isoflurane exposure. A novel finding of our study is that isoflurane may induce NLRP3 inflammasome activation of the primed microglial cells. This result suggests that NLRP3 priming might be a condition for isoflurane to induce cognitive dysfunction and may be one of the reasons why elderly patients are more prone to POCD than young patients.

We showed that isoflurane induced age-related hippocampal neuroinflammation via NLRP3 inflammasome activation. We further showed this effect in a microglial cell line and primary microglial cultures. Of note, it is reported that many aspects of microglial signature are not present in microglial cell lines [45]. However, studies have reported that the BV-2 cell line and neonatal primary microglial cultures react in a similar way on NLRP3 inflammasome activation [46, 47]. Our preliminary work also found that the NLRP3-caspase-1 pathway could be regulated by different doses of LPS. Thus, we chose the BV-2 cells and neonatal primary microglial cultures for mechanistic study. Our results suggest a critical role of NLRP3 primed status in inducing mature proinflammatory cytokine production. We also showed that isoflurane activated NLRP3 inflammasome possibly by increasing the assembly of NLRP3 inflammasome. It is reported that a small amount of NLRP3 inflammasome complex is assembled but remains in resting status under control condition. The activation process recruits additional members and activates caspase-1 [17]. The detailed mechanism for isoflurane to induce NLRP3 inflammasome activation needs to be investigated in the future studies.

We have shown that isoflurane induces learning and memory dysfunction in elderly rats and that this effect is IL-1β-mediated [13, 15]. In this current study, we showed that isoflurane-induced impairment of learning and memory is age-dependent. This phenomenon may be due to the primed status of microglia in the aged brain. Specifically, we found that the NLRP3 inflammasome might contribute to isoflurane-induced neuroinflammation and cognitive impairment in the aged mice. These findings advance our understanding on how isoflurane induces neuroinflammation and cognitive dysfunction and suggest that NLRP3 inflammasome may be a therapeutic target for POCD.

Our study has limitations. First, we did not perform any surgical stimulation during the isoflurane exposure. The surgical effects on NLRP3 inflammasome activation are not known. Second, we only used male mice in this study to reduce the potential influence of fluctuating estrogen and progesterone concentrations in learning and memory in female mice. Thus, the role of NLRP3 activation in isoflurane effects on female animals is not known. Third, we did not study cell viability in depth after isoflurane exposure because it is not a focus of our current study. However, we showed that isoflurane did not change the viability of the BV-2 cells and primary microglial cultures and did not induce apoptosis in the hippocampus, indicating that isoflurane-induced, age-related cognitive impairment may be independent of changes in brain cell viability. Fourth, there are differences between adult microglia in the brain and BV-2 cells or neonatal primary microglial cultures [45]. The in vivo system is very complex, and it may be difficult to use in vivo system to dissect cell type-specific effects. In addition, it has been reported that BV-2 cells and neonatal primary microglia cultures react in a similar way on NLRP3 inflammasome activation to that under in vivo condition [47, 48]. Also, NLRP3 may mainly be expressed in the microglia [42, 49]. Thus, our results suggest the role of microglial activation in the effects we observed here.

In summary, we have shown that aged mice had learning and memory impairment after isoflurane anesthesia, and this isoflurane effect was not apparent in young mice. The impairment in the aged mice was reversed by the inhibition of NLRP3-caspase-1 pathway. Isoflurane also induced age-related hippocampal inflammation through NLRP3-caspase-1 pathway. NLRP3 inflammasome priming is necessary in isoflurane-induced IL-1β production. These results suggest that isoflurane induces age-related cognitive dysfunction via NLPR3 activation.

## Conclusions

NLRP3 priming status in aged mouse brain may be involved in isoflurane-induced hippocampal inflammation and cognitive impairment.

## Additional file

Additional file 1: Supplementary material for NLRP3 priming induced by LPS stimulation. (DOCX 90 kb)

## Abbreviations

AC: Aged mice not being exposed to isoflurane or any drugs
A-ISO: Aged mice exposed to isoflurane
A-ISO-cmk: Ac-YVAD-cmk administered before aged mice were exposed to isoflurane
A-ISO-PBS: Solvent administered before aged mice were exposed to isoflurane
ANOVA: Analysis of variance
ASC: Apoptosis associated speck-like protein containing a caspase recruitment domain
CCK-8: Cell Counting Kit-8
CNS: Central nervous system
DAMPs: Damage-associated molecular patterns
DMSO: Dimethylsulphoxide
ELISA: Enzyme-Linked Immunosorbent Assay
FBS: Fetal bovine serum
GAPDH: Glyceraldehyde-3-phosphate dehydrogenase
IL-1β: Interleukin -1β
IL-18: Interleukin − 18
LPS: lipopolysaccharide
MWM: Morris water maze
NF- κB: Nuclear factor-κB
NLRP3: NOD-like receptor protein 3
PBS: Phosphate-buffered saline
POCD: Postoperative cognitive dysfunction
TNF-α: Tumor necrosis factor
YC: Young mice not being exposed to isoflurane or any drugs)
Y-ISO: Young mice exposed to isoflurane
