# Supplementary material for: Critical role of NLRP3-caspase-1 pathway in age-dependent isoflurane-induced microglial inflammatory response and cognitive impairment
Source: J Neuroinflammation. 2018 Apr 17;15:109. doi: 10.1186/s12974-018-1137-1 (PMC5904978; doi:10.1186/s12974-018-1137-1)
Supplement: Supplementary file 1 — Supplementary material for NLRP3 priming induced by LPS stimulation. (DOCX 90 kb) [file 12974_2018_1137_MOESM1_ESM.docx]

**
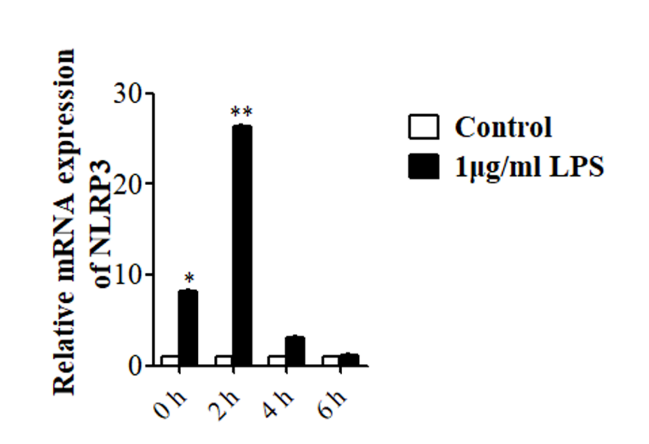
**

**Figure legend. NLRP3 priming was induced by LPS stimulation.** LPS at 1 μg/mL was added to the cell culture media of BV-2 cells for 30 min. NLRP3 mRNA was quantified by real-time qPCR 0 h, 2 h, 4 h and 6 h after LPS stimulation. Values are expressed as fold changes over the mean values of blank control. All results are presented as mean ± S.D. (n ≥ 3). * P < 0.05, ** P<0.01 compared with the corresponding data of blank control.
